# Teaching Research Data Management with DataLad: A Multi-year, Multi-domain Effort

**DOI:** 10.1007/s12021-024-09665-7

**Published:** 2024-05-07

**Authors:** Michał Szczepanik, Adina S. Wagner, Stephan Heunis, Laura K. Waite, Simon B. Eickhoff, Michael Hanke

**Affiliations:** 1 Institute of Neuroscience and Medicine, Brain and Behaviour (INM-7), Research Center Jülich, Jülich, Germany; 2 Institute of Systems Neuroscience, Medical Faculty, Heinrich Heine University Düsseldorf, Düsseldorf, Germany

**Keywords:** Research data management, Version control, Online course, Software documentation, Tutorial, Workshop

## Abstract

Research data management has become an indispensable skill in modern neuroscience. Researchers can benefit from following good practices as well as from having proficiency in using particular software solutions. But as these domain-agnostic skills are commonly not included in domain-specific graduate education, community efforts increasingly provide early career scientists with opportunities for organised training and materials for self-study. Investing effort in user documentation and interacting with the user base can, in turn, help developers improve quality of their software. In this work, we detail and evaluate our multi-modal teaching approach to research data management in the DataLad ecosystem, both in general and with concrete software use. Spanning an online and printed handbook, a modular course suitable for in-person and virtual teaching, and a flexible collection of research data management tips in a knowledge base, our free and open source collection of training material has made research data management and software training available to various different stakeholders over the past five years.

## Introduction

While experts in their respective domains and methodologies, scientists may not have domain-agnostic technical skills which are useful for efficient research data management (RDM). Managing the life cycle of digital objects which constitute research data requires a broad set of technical skills, however, research curricula seldom teach computing ecosystem literacy (Grisham et al., 2016). In fact, even computer science curricula often miss critical topics about the computing ecosystem. At the Massachusetts Institute of Technology (MIT), USA, this lack famously resulted in the internationally popular, self-organized class, “The missing semester of your CS education”^1^. In addition, the high usability of modern computers’ and applications’ front ends spares users the need to develop the same level of familiarity with their computers that previous generations of computer users had (Mehlenbacher, 2003). Yet, making research data and results findable, accessible, interoperable, and reusable (FAIR, Wilkinson et al., 2016) can benefit, among others, from efficient use of various research software tools (Wiener et al., 2016). This makes general technical skill and RDM training a crucial element in preparing the next generation of neuroscientists.

In an ongoing multi-modal, multi-year effort, we combined various interconnected activities into a comprehensive RDM training centered around the software tool DataLad (datalad.org; Halchenko et al., 2021). These activities spanned a community-led online RDM handbook with a printed paperback option and knowledge base, a matching online RDM course, and various workshops. In this reflective piece, we evaluate this teaching ecosystem, review its advantages and shortcomings, and share lessons learned over its 5-year long history.

### DataLad

DataLad is a Python-based, MIT-licensed software tool for the joint management of code, data, and their relationship. It builds up on git-annex, a versatile system for data logistics (Hess, 2010), and Git, the industry standard for distributed version control. To address the technical challenges of data management, data sharing, and digital provenance collection, it adapts principles of open-source software development and distribution to scientific workflows. Like Git and git-annex, DataLad’s primary interface is the command line. This makes familiarity with the computer terminal, common file system operations, and general knowledge about one’s operating system beneficial for software users.

### Overarching Goals for Training Materials

Our training material aims to provide even technical novices with the opportunity to use the software quickly, productively, and to easily integrate with other tools and services in real-world research. In part, it was motivated by concrete user needs, such as early career researchers in a research consortium. Beyond this, we aimed for the training material to be fully open source, accessible (both regarding language and technical requirements), flexible, multi-modal (everyone should find something that fits their learning needs), directly applicable to various research contexts, and maintainable.

## A DataLad Research Data Management Handbook

Since the first release (0.0.1, March 2015), DataLad had technical documentation with a design overview and reference documentation. Although any amount of documentation is better than no documentation at all, existing documentation can still be insufficient if it does not meet the needs of the target audience. Solely technical or reference documentation, for example, can be suboptimal for novices: it may be incomplete, narrowly focused on individual commands, or assume existing knowledge readers lack (Segal, 2007; Pawlik et al., 2015), and can thereby discourage potential users or inhibit the adoption of a tool. Even though technical documentation is useful for developers, a central target audience for documentation of the DataLad ecosystem are scientists. A considerable part of this target audience can thus be considered technical novices for whom technical documentation is not ideal. Research also suggests that scientists need documentation to go beyond reference manuals. In an analysis of user questions in support forums of scientific software packages, Swarts (2019) found that the focus in 80% of inquiries was on operations and tasks, such as a required sequence of operations to achieve a specific goal, instead of reference lists. In breaking down user questions by purpose, Swarts (2019) further found that users were most interested in a description of operations or tasks, followed by insights about the reasons behind the action. Separating documentation types into *feature-based* (closer related to the concept of reference documentation) or *task-based*, Swarts (2019) reports twice as many questions seeking explanations in software with *feature-based* compared to *task-based* documentation. This hints at a disconnect between knowing *how* something should be done and *why* it should be done this way. Overall, this highlights that users of scientific software show a clear need beyond the documentation of individual commands, but seek to understand general usage principles and master complex combinations of features to achieve specified goals. This type of empowerment is what the DataLad Handbook project aimed to achieve by complementing DataLad’s existing technical documentation.

### Design Considerations

We identified three types of stakeholders with different needs: researchers, planners and trainers. *Researchers* need accessible educational content to understand and use the tool; *planners*, such as principal investigators or funders, need high-level, non-technical information in order to make informed yet efficient decisions on whether the tool fulfills their needs; and *trainers* need reliable, open access teaching material. Based on this assessment, the following goals for the Handbook’s contents were set:

- **Applicability for a broad audience**: The Handbook should showcase domain-agnostic, real-world RDM applications.
- **Practical experience**: The Handbook should enable a code-along style usage, with examples presented in code that users can copy, paste, and run on their own computer. To allow a read-only style usage, too, the Handbook should also reveal what a given code execution’s output would look like. For an optimal code-along or read-only experience, the code output should match the current software behavior.
- **Suitable for technical novices**: The Handbook’s language should be accessible. Gradually, by explaining technical jargon and relevant tools or concepts in passing, it should provide readers with a broad set of relevant RDM skills rather than requiring prior knowledge.
- **Low barrier to entry:** The Handbook’s contents should be organized in short, topical units to provide the possibility to re-read or mix and match.
- **Integrative workflows**: The Handbook’s contents should build up on each other and link back to content already introduced to teach how different software features interact.
- **Empowering independent users**: Instead of showcasing successful code only, it should also explicitly demonstrate common errors to enable users to troubleshoot problems in their own use cases independently.

The following structure arose from this specification analysis (Wagner et al., 2020):

Introduction: The first part of the Handbook, covering high-level descriptions of the software and its features and detailed installation instructions for all operating systems.
Basics: The second part of the Handbook, written in the form of a continuous, code-along tutorial, set in a domain-agnostic fictional storyline about an RDM application, and covering all stable software features in chapters that build up on one another.
Advanced: The third part of the Handbook covering features beyond the basics in stand-alone chapters, added prior to the second release.
Use cases: The last part of the Handbook, containing short, standalone start-to-end descriptions of real-world use cases, with concise step-by-step instructions, and references to further reading in the Basics part.

Finally, the design and content requirements were accompanied by technical goals: from using expandable details to keep visible "core" text short and making the Handbook available in multiple formats, to developing the Handbook alongside the versioned software and using integration tests to ensure functioning of included code examples. The resulting implementation of the Handbook fulfilled these requirements as follows.

### The Technical Backbone

The development environment of the Handbook was chosen with the intent to support declared goals, and to maximize configurability, autonomy, and reusability of the project. It builds up entirely on flexible and extendable open source infrastructure: on the highest level, it uses Sphinx as a documentation generator (sphinx-doc.org). Sphinx transforms documents written in reStructuredText, a lightweight markup language, to a variety of output formats, among them HTML, PDF, LaTeX, or EPUB. Initially a by-product of the Python documentation, it has been adopted by the Open Source community at large; GitHub’s dependency graph reports that it is used by more than 300.000 projects in January 2024^2^.

**Fig. 1:**
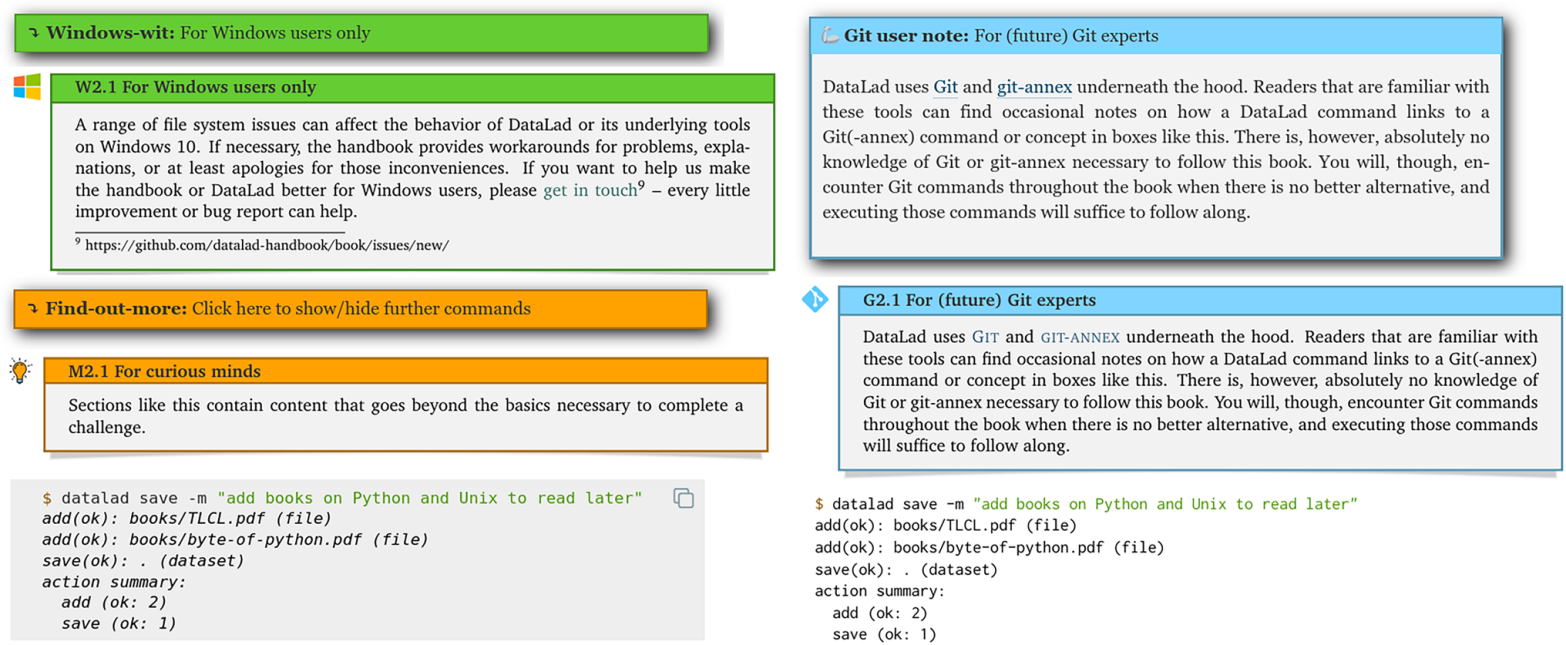
Custom admonitions and code blocks used in the Handbook. In each pair of admonitions, the top image corresponds to the web version, and the bottom image corresponds to its PDF rendering. Windows-wits (green), toggle-able in the HTML version, contain information that is only relevant for the Windows operating system (DataLad supports GNU/Linux, MacOS, and Windows, but the latter is fundamentally different compared to the other two, sometimes leading to different behaviour or necessitating workarounds when using DataLad). Find-out-more admonitions (orange), also toggle-able in the HTML version, contain miscellaneous extra information for curious readers. Git user notes (blue) are colored boxes with references to the underlying tools of DataLad, intended for advanced Git users as a comparison or technical explanation. Code blocks show one or more commands and the resulting output, provided using the autorunrecord Sphinx extension. In the web version, a copy-button (top right corner) allows to copy relevant commands automatically to the clipboard. Internal annotations allow generating custom scripts from any sequence of code-blocks for live coding demonstrations

Sphinx supports an extension mechanism with which additional functionality can be integrated. Leveraging this mechanism, the Handbook project extended standard Sphinx features with custom admonitions and designs, for example toggle-able boxes for optional details. This is implemented as a Python package alongside the Handbook source code, making the Handbook project a reusable and installable Sphinx extension. Figure 1 provides an overview of the custom-developed design features. A major functional enhancement is provided with a separate Python package, autorunrecord, an additional custom-made Sphinx extension that allows sequential execution of code in a specified environment, and embedding a record of the code and its output as code snippets into the documentation^3^. Instructors can further use it to automatically create scripts from selected code blocks which can then be demonstrated in a remote-controlled terminal in live-coding tutorials.

Hosting for the project is provided by Read the Docs (readthedocs.org), a full-featured software documentation deployment platform that integrates with Sphinx. Notably, it supports semantic versioning of documentation, which helps to ensure that users of a past software version can find the corresponding version of the conjointly developed Handbook. Illustrations in the Handbook are based on the undraw project by Katerina Limpitsouni (undraw.co).

The ability of the documentation to sequentially execute code and record its outcomes allows using the Handbook as an integration test for the DataLad software in addition to a user guide. If new software developments in the DataLad core packages break documented workflows, a continuous integration test suite will fail, alerting developers to the fact that their changes break user workflows.

To ensure reusability, such as the adaptation by Brooks et al. (2021), the project is released under a CC-BY-SA 4.0 license. Under its terms, all elements can be reused in original or derived form for all purposes under the condition that the original project is attributed and that derivative work is shared under an identical (“not more restrictive”) license^4^.

### Content

As of January 2024, the web and PDF versions of the Handbook were organized into four parts – “Introduction”, “Basics”, “Advanced”, and “Use cases” – which comprised a total of 21 chapters. The “Introduction” part has two different target audiences: first, it provides *researchers* with detailed installation instructions, a basic general command line tutorial, and an overview of the Handbook. Beyond this, it gives a high-level overview of the software and its capabilities to *planners*.

The “Basics” part is organized into nine chapters. Following a narrative about a fictional college course on RDM, it teaches different aspects of DataLad functionality and general RDM to *researchers* in each topical chapter. Broadly, those topics can be summarized as follows: 1) Local version control, 2) Capturing and re-executing process provenance, 3) Data integrity, 4) Collaboration and distributed version control, 5) Configuration, 6) Reproducible data analysis, 7) Computationally reproducible data analysis, 8) Data publication, and 9) Error management.

The “Advanced” part includes independent chapters on advanced DataLad features and workflows, big data projects, DataLad use on computational clusters, DataLad’s internals, and selected DataLad extensions. The latter two parts are accompanied with code demonstrations, slides, executable notebooks, and/or video tutorials that *trainers* can reuse freely to teach tool use and improve scientific practice. The last part, “Use cases”, targets *planners* and *researchers* with short step-by-step instructions which show *planners* what is possible, and help *researchers* to connect their knowledge into larger workflows.

### Project and Community Management

Ensuring the longevity of software projects beyond the duration of individual researchers’ contracts requires community building (Koehler Leman et al., 2020). A user-driven alternative to documentation by software developers, “Documentation Crowdsourcing”, has been successfully employed by the NumPy project (Pawlik et al., 2015). The Handbook project extends this concept beyond reference documentation. To achieve this, it is set up to encourage and welcome improvements by external contributors. The project is openly hosted on GitHub. Mirroring processes in larger crowd-sourced documentation projects such as “The Turing Way handbook for reproducible, ethical and collaborative research” (The Turing Way Community, 2022), credit is given for both code-based and non-code-based contributions. Contributors are recognized in the source repository, on the DataLad Website, and as co-authors in both the printed version of the Handbook and its Zenodo releases. As of January 2024, a total of 60 contributors provided input in the form of content, bug fixes, or infrastructure improvements.

### Paperback Version

A digest of the Handbook was published via the Kindle Direct Publishing (KDP) print-on-demand service to make the Handbook available in a printed paperback version. This fulfilled user demands for physical copies of the documentation, and was possible with minimal additional technical work, building up on the automatically generated LaTeX sources of the Handbook. The printed book’s contents were sub-selected for longevity, graphics or graphical in-text elements were optimized for black-and-white printing, and a dedicated hyperlink index was created.

## RDM Online Course

While documentation is the primary way of disseminating information about software, workshops are another often practiced way of software education. As maintainers and contributors of DataLad, we receive invitations to teach such workshops for different audiences, most commonly involving early career researchers. Some such events arise from obligations related to consortium participation (such as the CRC 1451^5^, Collaborative Research Center, investigating mechanisms of motor control, where RDM training was organised with course credit for involved doctoral students); others stem from more informal collaborations. To be better prepared for organizing training events, we decided to create a curriculum for a short RDM course centered around DataLad^6^. Our design approach aligns with the “Ten simple rules for collaborative lesson development” (Devenyi et al., 2018), and the course content and format were inspired by the Software Carpentry courses (Wilson, 2016).

### Design Considerations

While the Handbook is meant to be a comprehensive set of documentation covering multiple aspects, the course materials were intended as a more focused overview of the key features of DataLad software; self-contained, but linking to the Handbook for detail or context when needed. They introduce DataLad via interdependent examples which help present both usage and purpose of its basic commands. While the course focuses on DataLad, software-independent information about good practices in research data management is also included. The structure was tuned for presentation during a hands-on workshop (online or in-person) as well as self-study. The intended workshop duration was two half-days. Making it easy for tutors (also those who were not involved in course preparation) to reuse the materials on different occasions was an important goal; as time constraints and target audiences can differ, contents were divided into four core blocks and two optional additions. Finally, the aim was to create an open resource, not just by publishing the materials as a public website, but also by hosting the collaboratively edited sources in a public repository, licensing the content under Creative Commons Attribution License, and reusing other permissively licensed materials.

During the planning phase, we identified a set of data management tasks which should be covered, from dataset creation and local version control, through data publishing in external repositories and collaboration, to reusing datasets published by others and creating an analysis with modular datasets. The major theme for the software-agnostic part about good RDM practices, which came up in an informal poll among our colleagues, was file naming and organisation. Although it may sound trivial at first glance, this includes topics such as rationales for naming schemes, interoperability considerations related to file names (lengths, character sets), avoiding leakage of identifying information through file names, using sidecar files for metadata, clear semantics for separating inputs and outputs, and standard file organization structures, e.g. BIDS (Gorgolewski et al., 2016) or research compendium (Gentleman & Temple Lang, 2007). In addition to these, we also decided to discuss the distinction between text and binary files, and show examples of how the former can be used to store different kinds of data and metadata in an interoperable fashion (tabular files, serialization formats, lightweight markup).

### The Technical Backbone

The course website with the full course material is created based on The Carpentries^7^ lesson template^8^. Website content is written in Markdown, and the website is built with the Ruby-based static site generator Jekyll (note, however, that The Carpentries recently redesigned their tooling to use R’s publishing ecosystem instead^9^). Course material is split into sections, each starting with an overview (questions, objectives, time estimate), and ending with a summary of key points. The content is presented using a combination of text paragraphs and template-defined boxes with code samples, expected output, call-outs, challenges, and more.

During courses, we use Jupyter Hub to provide a unified, pre-configured software environment for participants, accessible through a web browser. While Jupyter Hub is mainly associated with notebooks, we mostly use its terminal feature, effectively providing participants with browser-based access to a terminal running on a remote machine. To simplify deployment, we use The Littlest JupyterHub^10^ (TLJH) distribution to set up the hub for all users on a single machine. We have used Amazon Web Services to provision virtual machines, but other cloud computing providers or local infrastructure can be used to the same effect. Setup instructions, expanded from TLJH’s documentation, were included in the course website, in the “For instructors” section.

### Content

Following the design considerations, we organized the course in the following modules:

- **Content tracking with DataLad**: learning the basics of version control, working locally to create a dataset, and practicing basic DataLad commands.
- **Structuring data**: listing good practices in data organization, distinguishing between text and binary data, and exploring lightweight text files and how they can be useful.
- **Remote collaboration**: exercising data publication and consumption, and demonstrating the dissociation between file content and its availability record.
- **Dataset management**: demonstrating dataset nesting (subdatasets), investigating structure and content of a published dataset, and creating a simple model of a nested dataset.
- (optional) **The basics of branching**: understanding Git’s concept of a branch, creating new branches in a local dataset and switching between them, and mastering the basics of a contribution workflow.
- (optional) **Removing datasets and files**: learning how to remove dataset content, and removing unwanted datasets.

Additionally, the course website contains a short glossary, setup instructions for users (if using their own computers), slides, and instructor notes about the technical setup.

## Knowledge Base and Online Office Hours

The educational resources were designed to be broadly applicable and domain-agnostic, but could not necessarily cover arbitrary use cases. Practical application of DataLad in RDM scenarios involves developing solutions to complex problems. No individual solution will always be one-size-fits-all, and no documentation can ever be comprehensive for everyone without becoming overwhelming for some. Likewise, while useful for discovering and learning usage patterns, most resources were of limited utility for trouble-shooting software issues. To this end, we offer a weekly online office hour to provide flexible assistance, and we invested resources into creating a knowledge base^11^. Office hours are a one-hour open video call during which (prospective) users can join flexibly and without prior notice, and ask questions or discuss use cases, often live-demoing relevant information via screen-sharing. The knowledge base, on the other hand, is a collection of documents, each document focusing on a particular topic (application, problem, solution) and considered standalone with respect to other documents. The nature of these documents resembles technical reports, the creation of which has a long-standing tradition in science and engineering (Pinelli et al., 1982; Brearley, 1973). When explorations in an office hour uncover technical limitations requiring workarounds or interesting use cases that are too peculiar to be prominently documented in the Handbook or RDM course, they typically inspire an entry in the knowledge base.

### Design Considerations

A knowledge base provides resources to anyone seeking particular solutions. It can also be used to accumulate the outcomes of investigations of technical issues as they occur when supporting users, thereby yielding persistent resources that streamline future support efforts, and increasing the efficacy of resources invested in support (turning incoming feedback into knowledge). An analysis of the content written on-demand, or its access frequency can also be used to inform prioritization of development efforts to improve technical implementations and/or documentation elsewhere.

Suitable topics for a knowledge base item (KBI) include: an answer to a frequently asked question (be that from office hours, issue trackers, or community forums); tips and strategies for a particular use case; a description of a technical limitation and possible workaround. Each KBI needs to have: a descriptive title; metadata, such as keywords, to aid discovery; and a persistent URL to share it.

### The Technical Backbone

The technical framework for the knowledge base is a simplified version of that used for the Handbook. In summary, KBIs are plain-text documents with reStructuredText markup. All KBI files are kept in a Git repository. A rendered knowledge base in HTML format is created with the Sphinx tool. A knowledge base Git repository is managed with the aid of a Git hosting solution, such as GitHub/GitLab. Respective continuous integration and website publishing tools are used to publish the knowledge base. Coordination for the office hour is done using a public matrix^12^ chatroom, in which questions can also be asked asynchronously.

### Content

As of January 2024, the knowledge base contains 29 KBIs of varying length, describing various use cases. For example, the first KBI that we created describes a situation in which DataLad (or Git) users working on shared infrastructure can trigger Git’s safety mechanism, added to Git versions released after March 2022, which causes certain operations to end with an error message displayed to the user. The knowledge base format allowed us to explain in detail not only the configuration options that need to be set in order to perform the operation, but also the broader rationale for the safety mechanism being present in Git in the first place (quoting, e.g., the informative commit messages which accompanied the changes made in Git).

## Impact and Scope

### Online Handbook

Work on the Handbook began in June 2019, and the first release followed in January 2020. It has been under continuous development for more than four years, averaging two releases per year, and complements the DataLad ecosystem with a comprehensive user guide. Its PDF version spans more than 600 pages. Releases of the DataLad core package are coordinated with matching releases of the Handbook project, and past release versions remain accessible online.

Confirming observations from the literature (van Loggem & van der Veer, 2014), the conjunct development of user documentation has positive effects on software quality. As the writing process involved manual software testing, initial developments were accompanied by a higher discovery rate of software errors. This user-focused approach uncovers deficiencies of the technical documentation and API elements with suboptimal user experience. The workflow-based nature of demonstrations highlights API inconsistencies, and the integration test that the Handbook constitutes catches incompatibilities between the software and common usage practice. These documentation features facilitate software development, and had a major impact on the conjoint 0.12.0 release of DataLad (Jan 2020), the first with a matching Handbook release. The popularity data confirms a marked increase in downloads of the DataLad Debian package from this date onward^13^. In addition, differences in web traffic confirm that user documentation is in higher demand than the technical documentation. An analysis of visits to the web version of the Handbook from December 2022 to July 2023 revealed that handbook.datalad.org averaged 22 000 total page views per 30 days, compared to 6600 for the technical documentation at docs.datalad.org. In summary, the development of the DataLad Handbook had a measurable positive impact on the number of users, the popularity of the package, and the software quality.

### Workshops

**Fig. 2:**
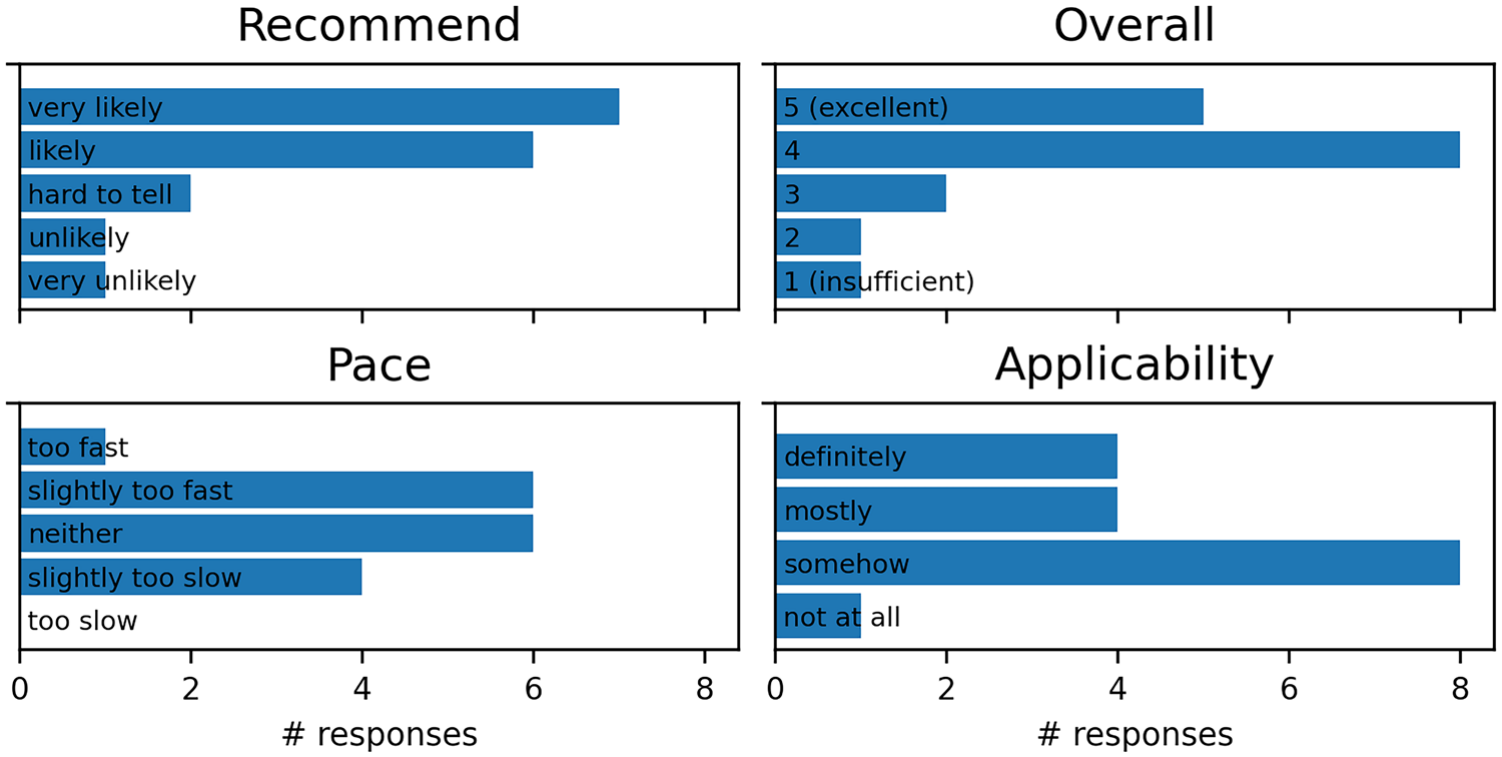
Responses of the participants of the first two installments of the workshop, conducted online for early career researchers, to the following questions. Recommend: How likely are you to recommend this workshop to a friend or colleague? Overall: What is your overall assessment of this event (1-insufficient, 5-excellent)? Pace: What do you think about the learning pace of the workshop (ie. material vs time)? Applicability: Will the knowledge and information you gained be applicable in your work?

We conducted a post-workshop survey among participants of the first two instances of the workshop conducted for the CRC 1451 early career researchers. Most participants were PhD students (who received course credit for workshop participation), and the workshops were conducted online. The workshop received high overall ratings, with participants stating that they are likely to recommend it to their colleagues; ratings of learning pace and applicability to participants’ work were mixed (Fig. 2). Given that the CRC 1451 project combines clinical, preclinical, and computational neuroscience, we see these responses as indicative of the diversity of backgrounds that PhD students in neuroscience have, as well as of a varying degree to which formal RDM is an already established practice across research fields. One recurring suggestion for improvement was to include more examples of real-world applications. This highlights that although a course dedicated to software basics is a good start, transferring the knowledge to specific applications is the real challenge, which can be made easier with existing written documentation.

### Knowledge Base

In the span of ten months we accumulated 29 knowledge base items of various length. The knowledge base has been useful in answering recurring questions, or communicating recommended workflows. Beyond this, it was also a valuable method to keep the official handbook and course material lean, as it provided a home to more temporary or niche use cases.

## Lessons Learned

In the previous sections we described the design considerations and their practical applications for the documentation and education aspect of the DataLad project. In our opinion, creating and maintaining this growing collection of materials was a worthwhile investment, which helped users to apply the software and developers to improve it. The open source, flexible approach to creating educational content was particularly valuable for its maintainability, adaptability, and applicability to various research contexts. In this closing section we want to share our comments – lessons learned in the process – on various related aspects.

### Customization vs Complexity

Our technical choices for the Handbook had to be weighed against non-technical features. Compared to other handbook projects such as the Turing Way project (The Turing Way Community, 2022), sources based on reStructuredText and Sphinx, as well as the many custom admonitions, constitute a higher barrier to entry for contributors. The Turing Way community, for example, explicitly chose Markdown-based Jupyter Book tooling to ease contributing for technical novices. Indeed, Handbook maintainers regularly have to assist new contributors with technical details, and complex technical contributions almost always come from the core contributor team. Nevertheless, in our case - especially with the requirements for multiple formats, integration tests, and reuse in print editions - the customization opportunities of Sphinx made up for slightly higher complexity than alternative documentation frameworks.

### Yes, there Can be too much Documentation

Although a large amount of documentation appears universally positive, there are concrete downsides that can increase with the amount of documentation. If more content leads to duplication, maintenance costs increase steeply, and so does the threat of showcasing outdated information. Thus, wherever possible, information is only detailed in a single location, and other places refer or link this source rather than duplicating its content.

Additionally, a large amount of documentation can appear intimidating. In our experience, information that educational resources exists is met positively, but the notion of a “600 page handbook” can diminish this enthusiasm. We find anecdotal evidence that a (surprisingly) large amount of available documentation can be perceived as a warning sign regarding software complexity, and is interpreted as a requirement to process all available documentation before a meaningful proficiency can be be reached. Designing all resources as best as possible in a modular, pick-what-you-need style proved to be important to allow for selective consumption and to lower the perceived cost of entry.

### Keeping Online Workshops Interactive

Keeping participants engaged during an online workshop is a particular challenge, as it is much harder to “read the room”. We have had positive experiences with using interactive poll and Q&A platforms, such as, e.g. DirectPoll^14^ or Slido^15^. Additionally, we believe that having co-presenters who can monitor text chat or take part of the questions is invaluable.

### Avoiding Installfest

Software should be easy to install, and we believe this is the case for DataLad. However, the preferred method of installation will differ between users. DataLad can currently be installed through several methods: conda, pip, apt and several other package managers (GNU/Linux), homebrew (MacOS). Selecting one of these methods will depend on how it integrates (or clashes) with the methods used for managing the entire software environment(s), and, if chosen hastily, may lead to future issues. To this end, we provide an overview of installation methods in the Handbook, and a note on debugging issues related to using multiple Python versions in the knowledge base. For this reason, performing an installation as part of the workshop may turn out to be time consuming, and we tend to avoid it. If installing a given software on participants’ computers is a goal (because it is required for the workshop or for future work), one approach that we found useful (at least with certain audiences) is to provide a link to detailed installation instructions beforehand and ask participants to e-mail the instructor with the output of a diagnostic command (or describe encountered problems). This encourages engagement from the participants, and may also provide instructors (maintainers) with an insight into how well the installation process works in practice.

### Software Environment Nuances

Exploring a command-line tool (particularly one for managing files) can hardly be separated from using basic command-line utilities (e.g. for changing the working directory or listing files). Although their usage can be weaved into the narrative of a workshop, this introduces additional complexity for command-line interface (CLI) novices. Moreover, while core utilities are similar across systems, there are differences, often subtle, in how they should be used to produce the same effect (e.g., compare *tree* vs *tree /F*, or *which* vs *Get-Command*, in Bash and PowerShell, respectively). The impact of these differences can be mitigated by providing toggle-able OS-specific instructions in the published materials, however, they still present a major challenge during live workshops when participants use different operating systems with their default sets of tools. For this reason, we prefer to use a common JupyterHub deployment for hands-on sessions.

### Cloud Computing

Both virtual and in-person workshops benefit from prepared virtual computing environments in particular. Costs per workshop amount to a few Euros, and typically never exceed 15 Euro even for multi-day workshops. The setup of the respective Amazon EC2 Cloud instance takes a few hours at most.

### Data Production and Data Consumption

The RDM course was created from the data producer perspective, and walks users through the process of building a dataset from scratch, covering data consumption only at a later stage. Aside from the fact that data analysis in computational neuroscience projects may just as often start with obtaining existing datasets, this narrative creates a situation where multiple steps are needed to reach a situation where benefits of version controlling the data can be seen. It could be an interesting change of perspective to start with obtaining a copy of an already created dataset, something which currently gets introduced in a later part of the workshop, and inspect its properties (such as content, history, and file availability information) in order to highlight the value added by RDM software. We tried this approach during shorter software demos, where having the target state communicated upfront was particularly useful for streamlining the presentation.

### Technical Writing Takes Time

Preparing a description of a discovered solution in the shape of a knowledge base item is time consuming, and a task on its own. However, it generates a resource which becomes useful with time, as the solution is captured with its context. Working on such solutions is a valuable way to learn about the program – also for developers who need not be familiar with all parts of the code base, or all potential applications.

## Conclusion

As the mere existence of software is insufficient to ensure its uptake and use according to best practices, maintaining user-oriented documentation became an important part of the DataLad project. Having users can not just validate the development effort, but it can also help enhance the software: users can diagnose problems, propose solutions, and suggest improvements (Raymond, 1999). A lack of documentation hinders knowledge transfer between users and developers, impedes maintenance, and creates a steep learning curve for new users and new developers alike (Theunissen et al., 2022). As described by Parnas (2011), “reduced [documentation] quality leads to reduced [software] usage, [r]educed usage leads to reductions in both resources and motivation, [r]educed resources and motivation degrade quality further”. Turning the argument around, improving documentation can improve software, which (for research software) can improve research.

Different kinds of documentation are needed for different audiences; in our case this led to creation of the Handbook, course materials, and knowledge base, in addition to the technical reference. In our interactions with users, we observe positive effects of having these resources available. We hope that our experiences in creating them, both in terms of design and practical aspects, can be helpful for other projects in research software development and for education in research data management.

## Information Sharing Statement

The sources of all projects described in this manuscript are available from GitHub and licensed under CC-BY, CC-BY-SA, or MIT licenses:

- github.com/datalad-handbook/book,
- github.com/datalad-handbook/book-datalad-intro,
- github.com/psychoinformatics-de/rdm-course,
- github.com/psychoinformatics-de/knowledge-base

## Data Availability

No datasets were generated or analysed during the current study.

## Footnotes

1 missing.csail.mit.edu

2 github.com/sphinx-doc/sphinx/network/dependents

3 github.com/mih/autorunrecord

4 creativecommons.org/licenses/by-sa/4.0

5 crc1451.uni-koeln.de

6 psychoinformatics-de.github.io/rdm-course

7 carpentries.org

8 github.com/carpentries/styles

9 carpentries.github.io/workbench/

10 tljh.jupyter.org

11 knowledge-base.psychoinformatics.de

12 matrix.org

13 qa.debian.org/popcon.php?package=datalad

14 directpoll.com

15 slido.com
